# The Single Toxin Origin of Alzheimer’s Disease and Other Neurodegenerative Disorders Enables Targeted Approach to Treatment and Prevention

**DOI:** 10.3390/ijms25052727

**Published:** 2024-02-27

**Authors:** Martin Tolar, John A. Hey, Aidan Power, Susan Abushakra

**Affiliations:** Alzheon, Inc., Framingham, MA 01701, USA

**Keywords:** Alzheimer’s disease, neurodegeneration, disease modification, beta-amyloid oligomers, protofibrils, APOE4, ALZ-801, valiltramiprosate, aducanumab, lecanemab, donanemab

## Abstract

New data suggest that the aggregation of misfolded native proteins initiates and drives the pathogenic cascade that leads to Alzheimer’s disease (AD) and other age-related neurodegenerative disorders. We propose a unifying single toxin theory of brain neurodegeneration that identifies new targets and approaches to the development of disease-modifying treatments. An extensive body of genetic evidence suggests soluble aggregates of beta-amyloid (Aβ) as the primary neurotoxin in the pathogenesis of AD. New insights from fluid biomarkers, imaging, and clinical studies provide further evidence for the decisive impact of toxic Aβ species in the initiation and progression of AD. Understanding the distinct roles of soluble and insoluble amyloid aggregates on AD pathogenesis has been the key missing piece of the Alzheimer’s puzzle. Data from clinical trials with anti-amyloid agents and recent advances in the diagnosis of AD demonstrate that the driving insult in biologically defined AD is the neurotoxicity of soluble Aβ aggregates, called oligomers and protofibrils, rather than the relatively inert insoluble mature fibrils and amyloid plaques. Amyloid oligomers appear to be the primary factor causing the synaptic impairment, neuronal stress, spreading of tau pathology, and eventual cell death that lead to the clinical syndrome of AD dementia. All other biochemical effects and neurodegenerative changes in the brain that are observed in AD are a response to or a downstream effect of this initial toxic insult by oligomers. Other neurodegenerative disorders follow a similar pattern of pathogenesis, in which normal brain proteins with important biological functions become trapped in the aging brain due to impaired clearance and then misfold and aggregate into neurotoxic species that exhibit prion-like behavior. These aggregates then spread through the brain and cause disease-specific neurodegeneration. Targeting the inhibition of this initial step in neurodegeneration by blocking the misfolding and aggregation of healthy proteins has the potential to slow or arrest disease progression, and if treatment is administered early in the course of AD and other neurodegenerative disorders, it may delay or prevent the onset of clinical symptoms.

## 1. Introduction

The past decade has brought remarkable advances in the diagnosis and understanding of the pathogenesis of Alzheimer’s disease (AD) and other neurodegenerative disorders, leading to the approval of the first wave of disease-modifying treatments. AD has become a model for the study of the origins and causes of brain neurodegeneration, from the use of fluid and imaging biomarkers tracking the progression of underlying pathologies to the application of such insights for successful drug development. Despite numerous studies evaluating a wide range of pharmacological treatments and drug mechanisms, only agents that block or prevent the formation of soluble beta-amyloid (Aβ, amyloid) aggregates called oligomers and protofibrils, or preferentially clear these species have shown clinical and biomarker efficacy in AD disease modification trials [1,2,3,4,5].

The Aβ peptide is a proteolytic derivative of the large transmembrane amyloid precursor protein (APP) and is formed by the sequential enzymatic cleavage of APP [6]. In its monomeric form, Aβ plays an important physiological role, protecting the human brain from injury, infection, and stress [7]. As a response to injury or stress, APP and Aβ production are upregulated, and Aβ concentrations, mostly Aβ40, increase acutely [8], providing the first-line response to infectious and toxic-metabolic brain insults. However, these responses, if left unchecked in the setting of deficient Aβ clearance mechanisms that occur with aging, can lead to Aβ accumulation and aggregation.

When brain concentrations of Aβ are elevated due to increased production as a result of genetic mutations, in response to injury or stressful conditions, or due to decreased brain clearance associated with aging, the monomeric Aβ peptides misfold and aggregate, gaining prion-like capabilities that seed further aggregation and propagation through brain structures [9]. Aβ peptides are inherently unstable and prone to misfolding and aggregation, especially the longer pathological Aβ42 species [10]. Aβ aggregation results in oligomers of many sizes, from dimers to dodecamers with molecular weights of 8–10 kDa to 60–80 kDa. Further Aβ aggregation yields larger oligomers with molecular weights of 80–500 kDa, called protofibrils, which are thought to be less toxic than the smaller oligomers. Misfolded Aβ propagates and spreads through the brain in a predictable and conserved pattern [10,11,12]. These oligomers and protofibrils inhibit long-term potentiation, the neuronal substrate for memory; cause neuronal stress and synaptic dystrophy; and trigger tau pathology that spreads along neuronal networks [11], ultimately leading to neuronal cell death [1,13]. The progressive synaptic failure and network dysfunction manifest as the progressive loss of cognitive abilities and executive function, described clinically as AD dementia.

One of the brain’s primary defenses against the toxicity of soluble oligomers is to sequester these neurotoxic species into insoluble amyloid fibrils and plaques, the pathognomonic histopathologic feature of AD [1,13]. Microglia, the immune cells of the brain, play a major role in the compaction of Aβ aggregates into insoluble and less toxic mature fibrils and dense plaques [14,15]. Additionally, perivascular astrocytes play a vital role in the clearance of Aβ aggregates into the glymphatic system and systemic circulation. At more advanced stages of the disease, microglia and astrocytes may also play a pro-inflammatory, harmful role in AD.

The primary risk factor for sporadic late-onset AD and many other neurodegenerative disorders is aging. In sporadic AD, the most common cause of the pathological increase in brain Aβ is impaired glymphatic clearance associated with aging due to either arteriosclerosis of the small brain vessels or the progressive dysfunction of the blood–brain barrier (BBB), affecting perivascular astrocytes and their aquaporin-4 water channels [16].

The second greatest risk factor for sporadic late-onset AD is the ε4 allele of apolipoprotein E (APOE4). APOE4 heterozygotes have an approximately four-fold increased risk of AD, whereas APOE4/4 homozygotes have a 10-fold to 12-fold increased risk compared with those with the neutral APOE3/3 genotype. The APOE4 genotype in AD is associated with accelerated Aβ aggregation, impaired Aβ clearance and an earlier age of AD onset due to insufficient uptake and clearance of Aβ through the LRP1 receptor on microglia and perivascular astrocytes [17], leading to deficiency in both intracellular proteolysis and glymphatic clearance of Aβ. APOE4 carriers accumulate more amyloid pathology and do so at a faster rate than non-carriers [18,19,20]. Recent proteomic analysis confirmed that APOE4 carriers have prominent dysfunction of the BBB [21].

Similarly, familial AD is caused by an increase in the production of Aβ due to mutations in APP or presenilin 1 or 2, leading to a very early onset of AD brain pathology and clinical symptoms in the fifth, fourth, or even third decade of life [22]. Another genetic form of AD is Down syndrome (DS) dementia, caused by the presence of the APP gene located on each chromosome 21, which is triplicated in DS. As a result, Aβ production is increased in DS, and characteristic AD brain pathology and biomarkers, as well as progressive cognitive impairment, are observed in most individuals with DS by the age of 40 [23,24].

AD has a very long preclinical phase, with a gradual accumulation of amyloid-driven pathology over approximately 20 years [25], followed by the phosphorylation of neuronal cytoskeletal microtubule protein tau. Elevated phosphorylated tau protein (p-tau) in the cerebrospinal fluid (CSF) or plasma is a marker of neuronal stress and a harbinger of incipient symptomatic AD.

**Figure 1:**
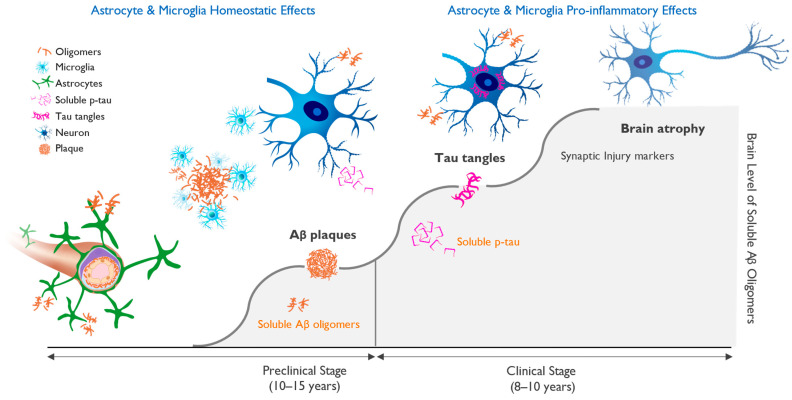
Progression of molecular pathology and neuronal dysfunction leading to clinical Alzheimer’s disease (AD). Clinical AD is preceded by a long silent pre-symptomatic phase. The accumulation of beta-amyloid-driven pathological changes in the brain occurs over 15–20 years and starts with the misfolding and aggregation of amyloid monomers into neurotoxic soluble oligomers, followed by neuronal dysfunction and cognitive impairment. Amyloid plaques serve as a protective brain mechanism, but once the ability to sequester beta-amyloid (Aβ) oligomers into insoluble fibrils and plaques is saturated, the oligomer toxicity triggers progressive neuronal stress with hyperphosphorylation of tau and the appearance of aggregated tau in neuronal cell bodies. This process correlates with markers of neuronal injury, eventually leading to neuronal cell loss and brain atrophy and the appearance of cognitive deficits. Figure 1 illustrates the importance of early diagnosis and intervention, ideally in the preclinical phase, in which treatment may allow for the maintenance of brain health and normal brain function.

Soluble tau “seeds” behave like prions and spread along synaptic networks, leading to their dysfunction. Soluble tau eventually aggregates into neurofibrillary tangles inside neurons, the second pathological hallmark of AD. This cascade of events, which is driven by increasing concentration of amyloid oligomers, is shown in Figure 1.

Amyloid and tau pathologies are the two defining features of AD, and the current biological basis of AD diagnosis for clinical trials requires positive amyloid and tau confirmation [12]. The earliest clinical stage of AD is defined as mild cognitive impairment (MCI), which progresses into mild AD once functional deficits appear several years later.

Therapeutic agents that block the formation of amyloid oligomers, e.g., small molecule ALZ-801/valiltramiprosate, or facilitate their removal, e.g., the approved and late-stage anti-amyloid antibodies aducanumab, lecanemab, and donanemab, have shown significant positive effects on AD biomarkers in clinical trials [2,26]. These anti-amyloid antibodies have also shown significant clinical efficacy in Phase 2 and 3 trials in early AD subjects [3,4,27,28], providing strong support for the primary role of oligomers in the pathogenesis of AD and for the validity of oligomers as a therapeutic target [13]. Oral ALZ-801/valiltramiprosate and its active agent tramiprosate have shown promising efficacy in APOE4 carriers in clinical trials [26,29], and oral ALZ-801/valiltramiprosate is currently being evaluated in the APOLLOE4 Phase 3 trial in APOE4/4 homozygotes with early AD.

## 2. Physiologic and Pathogenic Role of Beta-Amyloid Protein

Beta-amyloid is one of the most abundant proteins in both the peripheral and central nervous system (CNS), and it is estimated that the healthy adult brain contains 1.7 mg of Aβ or 5 µg/gm of grey matter [25]. Given its flexibility and ability to attach to other proteins, monomeric Aβ constitutes an important protective system in the brain that has been developed and preserved through evolution, beginning in species dating back hundreds of millions of years [7].

Soluble Aβ monomers exist as peptides of several lengths, cleave from APP located in the neuronal cell membrane, and, in the setting of impaired brain clearance mechanisms caused by aging or genetics, aggregate to form soluble Aβ oligomers and protofibrils of increasing molecular weight, ranging from the most toxic oligomers with a molecular weight of 10–80 kDa to less toxic protofibrils with a molecular weight of 80–500 kDa [6,30,31,32,33,34,35,36]. These soluble amyloid species further aggregate, ultimately forming insoluble mature fibrils and plaques.

The Aβ peptide is maintained in its native, unfolded monomeric form in the brain by several physiologic molecules, including 3-sulfopropanoic acid or 3-SPA, a metabolite of tramiprosate and an endogenous agent that has been shown to block amyloid misfolding, oligomer formation and aggregation [37].

Soluble Aβ monomers are produced continuously during neuronal activity and are cleared from the brain via an intricate system designed to maintain brain homeostasis. Physiological levels of soluble Aβ monomers in the brain are maintained by several active amyloid clearance mechanisms, including (1) extra-cellular proteolysis in interstitial fluid, (2) uptake and degradation by microglia and astrocytes, and (3) glymphatic clearance to the venous system facilitated by astrocytes [38,39]. The major pathway for amyloid removal from the brain is glymphatic clearance, which, when impaired in aging or in the presence of the APOE4 allele, plays a key role in the development of sporadic AD [40,41].

In addition, Aβ42 oligomers, which comprise the major fraction of soluble amyloid aggregates and are the most neurotoxic and synaptotoxic species, attenuate the protective effects of reelin, a major extracellular glycoprotein that supports synaptic function, particularly memory consolidation in the hippocampus and spatial learning [42,43,44]. The inhibition of this pathway leads to dyshomeostasis, a state of increased excitatory and diminished inhibitory processes with a net excitatory milieu that contributes to neuronal atrophy, the loss of dendritic spines, and ultimately neuronal death, resulting in progressive cognitive deficits.

The cerebrospinal fluid, which enters the perivascular space around penetrating cortical arterioles, diffuses into brain parenchyma driven by arterial pulsations, mixes with interstitial fluid (ISF), and carries solutes and protein waste toward the perivenular channels, called glymphatics, where the ISF exits the brain, flows into the meningeal lymphatic system, and drains into cervical lymphatics [45]. Perivascular astrocytes play a major role in Aβ clearance because their long extensions surround the microvessels and have aquaporin-4 water channels in their perivascular end-feet, channels that allow for the entry of water from the periarterial CSF into the brain parenchyma. Astrocytes facilitate the flow of ISF through the brain parenchyma, carrying waste products toward perivenous glymphatics and allowing their egress from the brain [16]. Perivascular astrocytes also carry LRP-1 receptors, which bind to soluble parenchymal Aβ, allowing its uptake and trafficking to astrocyte end-feet around capillaries, thereby promoting amyloid clearance to the plasma through the BBB. This process is deficient in APOE4 carriers [16,17].

The most abundant Aβ monomer in the brain is Aβ40, whereas the more toxic Aβ42 monomer is the species most prone to misfolding and aggregation and is the form most involved in the seeding of amyloid aggregates and oligomer formation. Aβ42 is also the major species found in insoluble plaque deposits in the human brain.

Small soluble Aβ oligomers are the most toxic amyloid species and consist of aggregates of two to twenty copies of Aβ monomers. These oligomers are highly neurotoxic and can further aggregate to form soluble large oligomers called protofibrils and then insoluble β-pleated sheets that form mature amyloid fibrils and plaques. As soluble Aβ42 is the predominant form sequestered into insoluble plaques, the ratio of soluble Aβ42 to Aβ40 in the CSF decreases early in AD, providing a useful diagnostic biomarker of the disease [46].

The evaluation of the ratio of soluble Aβ42 to Aβ40 can improve the accuracy of the diagnosis of AD and was found to correlate with positive amyloid positron emission tomography (PET) scans and changes in hippocampal volume [47]. The formation of insoluble fibrils and plaques appears to be a protective mechanism that mitigates and reduces oligomer toxicity [30,48]. At early stages of AD, microglia sequester soluble Aβ into plaques and enable phagocytosis of soluble species. Furthermore, microglia have been shown to play a critical homeostatic role in the AD brain by protecting the brain from injury and pathogens through a variety of neuroimmune actions, including the synaptic remodeling and trophic support of the brain parenchyma [14,49].

## 3. Amyloid Pathogenic Cascade

Our understanding of AD pathogenesis has advanced due to insights from longitudinal biomarker studies, brain amyloid and tau imaging, and large clinical trials of anti-amyloid agents. This has led to an updated clinical definition and staging of AD based on both amyloid and tau biomarkers and evidence of neuronal degeneration [12]. This A/T/N staging indicates that aggregation and accumulation of Aβ precedes and is required for the spread of tau pathology associated with the neuronal toxicity that leads to brain degeneration and atrophy.

The toxicity of soluble amyloid aggregates is the pivotal upstream trigger initiating the cascade of damaging molecular events, including tau hyperphosphorylation, neuroinflammation, calcium dysregulation, mitochondrial dysfunction, and oxidative stress, that cause progressive neurodegeneration and cell loss [50]. Small soluble Aβ oligomers, especially Aβ42 aggregates, are the leading cause of synaptic toxicity in AD [34,51,52,53,54]. Elevated brain Aβ oligomer levels are detectable in AD patients [53,55] and track closely with AD symptoms [33]. APOE4/4 homozygotes show increased brain concentrations of soluble Aβ oligomers, leading to earlier disease onset and a more aggressive course of AD [56,57].

**Figure 2:**
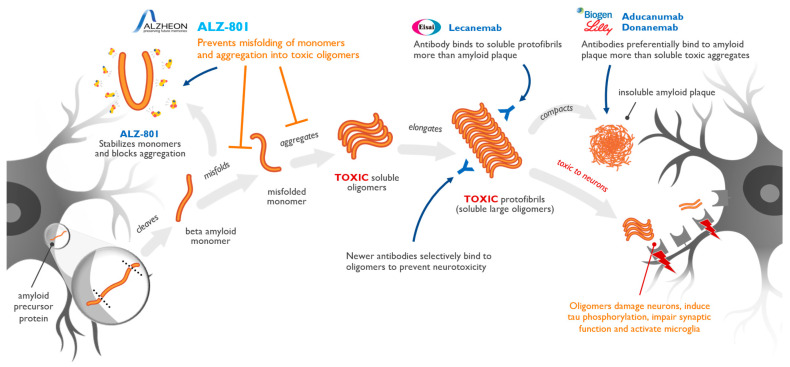
Alzheimer’s pathogenic cascade and impact of late-stage and approved anti-amyloid treatments. This schematic representation of the amyloid aggregation cascade describes the beta-amyloid (Aβ) selectivity of both the oral anti-oligomer agent ALZ-801/valiltramiprosate and injectable anti-amyloid antibodies that have shown clinical or biomarker efficacy in large clinical trials in AD. Mechanistic studies suggest that ALZ-801 acts upstream in the process and fully inhibits the pathogenic amyloid cascade, while antibodies demonstrate differential selectivity to already aggregated amyloid species and facilitate their removal from the brain. The collective body of evidence suggests that interaction with soluble aggregates drives clinical efficacy, while interaction with insoluble plaques is associated with brain edema and microhemorrhage side effects due to the removal of mature fibrils and plaques from the vessel walls, making them leaky for plasma and blood [1,2,13].

Figure 2 illustrates the pathogenic process of AD and the distinct impact of different amyloid species and highlights the molecular points of target engagement of anti-amyloid treatments that have shown disease-modifying effects in AD studies. Agents that have demonstrated positive clinical and biomarker effects include three anti-amyloid antibodies—aducanumab, lecanemab, and donanemab—and the small molecule inhibitor of amyloid oligomer formation, ALZ-801/valiltramiprosate. Aducanumab has received accelerated approval from the US Food and Drug Administration (FDA), and lecanemab has received both accelerated and traditional approvals from the FDA following the completion of a Phase 3 trial involving early AD subjects. Donanemab showed benefits in a Phase 3 trial involving early AD subjects [5], and ALZ-801/valiltramiprosate showed significant biomarker effects and promising clinical effects in a Phase 2 trial involving early AD subjects who were APOE4 carriers [26,58].

Each of these anti-amyloid antibody agents targets soluble oligomeric species with various binding profiles. ALZ-801/valiltramiprosate works upstream of antibodies in the pathogenic amyloid pathway, boosts endogenous protective brain mechanisms mediated by 3-SPA [37], and selectively inhibits the formation of oligomers through a novel enveloping mechanism of action that prevents the misfolding of amyloid [37,59]. Aducanumab, donanemab [9,60], and lecanemab [61] act further downstream, binding and removing toxic oligomers and protofibrils.

In the class of anti-amyloid antibodies, lecanemab has shown the best selectivity for soluble amyloid oligomers and the lowest selectivity for insoluble mature fibrils or plaques, resulting in a stronger efficacy and a better safety profile [61]. Interestingly, while the reported mechanism of action of donanemab is binding to plaques, a recent study showed that donanemab exhibits a profile of binding to the spectrum of soluble aggregated amyloid species overlapping that of aducanumab [9,60].

Amyloid plaques appear to play a protective role by sequestering Aβ oligomers and removing them from the local brain microenvironment [53,62]. Therefore, targeting Aβ plaques alone does not reduce the burden of toxic oligomers and, as such, does not confer clinical benefit, as shown with the anti-amyloid antibody gantenerumab, which has low specificity for soluble oligomers and which proved unsuccessful in Phase 3 trials [9,60,61,63].

## 4. APOE4 Represents Main Genetic Risk Factor for Alzheimer’s Disease

**Figure 3:**
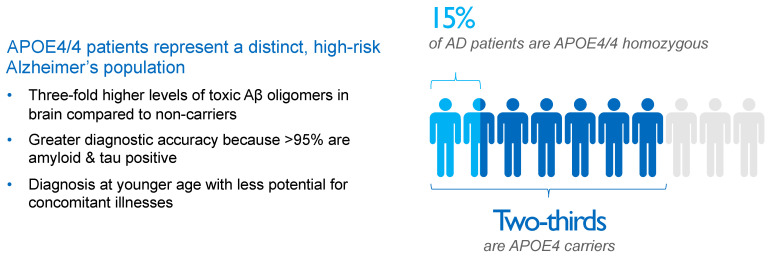
Impact and distribution of APOE4 genotypes in Alzheimer’s disease (AD) patients. APOE4 carriers represent approximately two-thirds of AD patients. APOE4/4 homozygotes, patients with the most aggressive form of sporadic AD, show several-fold higher concentrations of amyloid oligomers in the brain compared with non-carriers and an almost decade-earlier onset of the disease, thereby representing a suitable high-risk population to study the effects of anti-amyloid interventions. Aβ, beta-amyloid; AD, Alzheimer’s disease; APOE4, ε4 allele of apolipoprotein E gene; APOE4/4, homozygosity for ε4 allele of apolipoprotein E gene.

Compared to APOE4 noncarriers, the risk of AD is four-fold higher for APOE4 heterozygotes and 10-fold to 12-fold higher for APOE4/4 homozygotes [64] in populations based only on clinical diagnosis, rising to odds ratios of 4.6 and 25.4, respectively, for AD subjects diagnosed using CSF biomarkers [65]. As a result, approximately two-thirds of AD patients are carriers of the APOE4 gene, with roughly 50% being APOE4 heterozygotes and 15% being APOE4/4 homozygotes, as shown in Figure 3.

APOE is a lipoprotein that transports cholesterol and Aβ in the brain and plasma. Approximately 25% of individuals in the Caucasian population carry one APOE4 allele, and 2% to 3% of the population carries two alleles, but these APOE4 carriers comprise 65% to 70% of AD subjects in clinical trials utilizing a biomarker-based diagnosis of AD [65]. Approximately 10% to 15% of the 6.7 million AD patients in the United States are APOE4/4 homozygotes, with a similar number of APOE4/4 homozygotes with AD (~700,000) in the European Union [66,67].

As a result of increased brain Aβ concentration, APOE4 carriers develop more vascular amyloid pathology [68,69], called cerebral amyloid angiopathy (CAA). The deposition of amyloid in brain microvasculature and the resultant weakening of vessel walls underlies the occurrence of spontaneous brain edema and microhemorrhages observed on magnetic resonance imaging (MRI) scans [70], as well as the risk of brain edema and microhemorrhages following treatment with anti-amyloid antibodies [2]. These MRI lesions, when they occur via treatment with amyloid immunotherapies, are known as amyloid-related imaging abnormalities either with edema (ARIA-E) or with microhemorrhages and/or hemosiderin deposits (ARIA-H).

ARIA lesions were first described in the bapineuzumab trials [71], and, subsequently, they were reported in trials with other plaque-clearing antibodies [2,72,73]. APOE4/4 homozygous AD patients show the highest degree of CAA pathology [74] and also carry the highest risk of ARIA-E and ARIA-H when treated with anti-amyloid antibodies [2,73]. Therefore, there is an urgent unmet medical need for an agent that can deliver meaningful clinical efficacy in APOE4/4 homozygotes, without the increased risk of brain edema or microhemorrhage.

Since the oral anti-amyloid oligomer agent ALZ-801/valiltramiprosate inhibits the formation of Aβ oligomers without affecting plaques [59], ALZ-801 has the potential to be a suitable therapeutic for APOE4 carriers that does not increase the risk of ARIA.

## 5. Interaction of Aβ and APOE4 with Other Molecules in AD Brain

The interaction of Aβ species with various proteins in the AD brain is an area of increasing importance, and studies of the Aβ interactome have the potential to provide novel therapeutic targets [75]. One of the most studied protein interactions in AD is that of Aβ and APOE4 [76,77]. There are multiple mechanisms by which the APOE4 genotype confers increased AD risk, including decreased Aβ phagocytosis and clearance through the blood–brain barrier, increased tau hyperphosphorylation and aggregation, and exaggerated microglial and astrocytic responses. These effects collectively lead to diminished Aβ clearance, exaggerated neuroinflammation, and increased Aβ aggregation [78,79], all leading to increased levels of neurotoxic soluble Aβ oligomers and tau spreading in the brains of APOE4 carriers, especially APOE4/4 homozygotes [56]. Consistent with having the greatest amyloid burden and tau pathology, APOE4/4 subjects exhibit an earlier and faster rate of cognitive decline, becoming symptomatic approximately a decade earlier than non-carriers.

The interaction of APOE4 with the microglial activating receptor TREM2 is an emerging area of focus for drug discovery [80,81]. This is a complex relationship, with the effects of microglial activation being homeostatic and protective in the early stages of AD and pro-inflammatory and harmful at later stages. Therefore, TREM2-targeted drug development is challenging since the benefits of TREM2 activation may be specific to the disease stage.

Recent studies have highlighted the APOE4 interaction with the Reelin-Disabled-1 (Dab1) signaling pathway, which plays a protective role in synaptic development and plasticity [82]. Reelin signaling protects synapses from Aβ-induced neuronal stress, while APOE4 interferes with this protective effect, worsening synaptic toxicity of Aβ [83]. Genetic variations in the Reelin pathway have been described in a Spanish population to confer increased risk [84] and in the UK Biobank as risk factors for AD, particularly in APOE4/4 homozygotes [85]. A recent report described a protective Reelin variant in a male with autosomal-dominant AD [86].

## 6. Biomarkers and Biological Definition of Alzheimer’s Disease

The biological definition of AD has been essential for the success of drug trials with disease-modifying agents that target amyloid pathology, the only approach to date that has shown positive clinical and biomarker effects in delaying the progression of the disease. A clinical AD diagnosis without biomarker confirmation has very low accuracy in APOE4 non-carriers (~60%) and low accuracy in heterozygotes (80%) but has excellent accuracy in APOE4/4 homozygotes (>95%) [87].

New insights into the pathogenic role of Aβ and the application of brain biomarkers have markedly improved our ability to identify individuals with AD pathology long before the onset of clinical symptoms and have led to diagnostic criteria for clinical research based on objective disease biomarkers [12]. Data from longitudinal AD studies and interventional clinical trials using PET imaging of amyloid and tau aggregates and fluid biomarkers of AD were most helpful in advancing a biological definition of AD that avoids the pitfalls and substantial inaccuracy of reaching a clinical diagnosis, which was 30% in older clinical trials [12,87]. The onset of biomarker changes closely correlates with clinical onset and stages of AD and follows a well-defined sequence of pathological changes. An increase in brain Aβ concentration leads to amyloid accumulation into toxic soluble oligomers and protofibrils, initiating tau phosphorylation that can be detected by amyloid and tau PET scans. An increase in CSF or plasma biomarkers, including Aβ, p-tau, and neurofilament light chain protein (NfL), precedes brain volume loss that can be assessed by brain volumetric MRI and ultimately results in cognitive decline [88,89,90,91].

Biomarker-based AD diagnostic criteria hold the potential to enable the detection of pathological changes years and decades before the onset of clinical symptoms and require that subjects have positive amyloid and tau biomarkers, with or without evidence of neuronal injury or neurodegeneration. This diagnostic framework, called the A/T/N classification, is summarized in Table 1. A/T/N classification is based on the status of amyloid (A), tau (T), and neuronal pathology (N), as determined by amyloid and tau PET imaging or CSF or plasma biomarkers, including Aβ, p-tau-, NfL, and neurogranin, and by MRI measurements of brain volume loss focused on hippocampal volume and cortical thickness.

**Figure 4:**
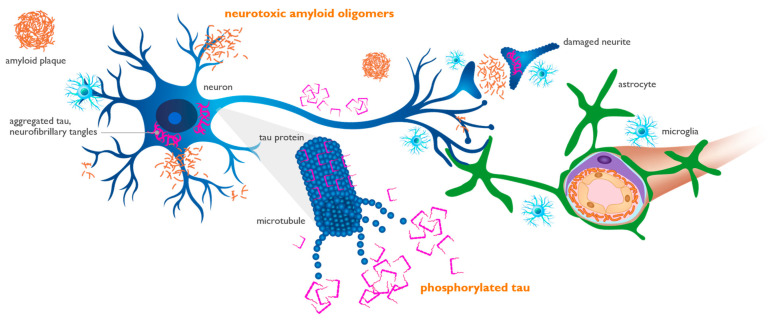
Amyloid oligomers induce synaptic dysfunction and neuronal injury, leading to the phosphorylation of tau protein. Amyloid oligomers injure synapses located on neuronal dendrites and cell bodies (blue cell). Phosphorylated tau (p-tau) is released from injured neurons into the interstitial fluid and can be measured in the cerebrospinal fluid and plasma, representing neuronal dysfunction and loss. Aggregated tau (purple) is shown as twisted neurofibrillary tangles within neurons, forming tau tangles. Perivascular astrocytes (green cell) play an active role in the trafficking and clearance of beta-amyloid (Aβ) and p-tau through the glymphatic perivascular system into systemic circulation, where they can be detected by plasma assays [88,89,91].

P-tau has recently emerged as the most reliable diagnostic and staging marker in AD. Tau, a cytoskeletal protein that forms the scaffolding of neurons called microtubules, is phosphorylated at threonine 181 or 217 sites when neurons are stressed and injured by toxic amyloid oligomers [46,88,89]. Longitudinal studies in AD subjects have shown that as levels of aggregated forms of amyloid increase in the brain, they induce abnormal phosphorylation of neuronal tau and a progressive elevation of p-tau in the CSF and plasma. These progressive increases in CSF and plasma p-tau precede the appearance of intraneuronal neurofibrillary tangles, the tau pathology that can be detected by tau PET imaging [90]. An increase in the concentration of toxic amyloid oligomers induces downstream synaptic dysfunction and neuronal injury, leading to the formation of p-tau, as shown in Figure 4.

P-tau_181_ and p-tau_217_ isoforms are found in the brain, CSF, and plasma of patients with AD pathology decades before clinical onset and have been shown to appear as a response to neurotoxic soluble amyloid aggregates [90,91]. These p-tau isoforms are specific to AD and serve as reliable biomarkers for tracking disease progression and for determining the impact of effective anti-amyloid therapeutics [88,89,91].

CSF assays for core AD biomarkers have been continually optimized, and some are approved for clinical use. More recently, plasma biomarker assays have also become sensitive and reliable for use in clinical trials. The late-stage amyloid antibody trials have included the evaluation of plasma biomarkers [3,4,94,95], focusing on p-tau_181_ and p-tau_217_ isoforms, which have been associated with efficacy on standard clinical endpoints. The effects of anti-amyloid agents on plasma p-tau_181_ and clinical outcomes are summarized in Table 2.

Anti-amyloid antibodies with meaningful clinical efficacy show plasma p-tau_181_ reductions of ≥15% from baseline over 1 year, while those that failed to achieve efficacy reduced p-tau_181_ by <10% over 1 year. This suggests that (1) the magnitude of plasma p-tau_181_ reduction over 1 year is a reasonable predictor of clinical efficacy and (2) early and sustained reduction in p-tau_181_ may be a suitable marker of target engagement and meaningful clinical efficacy. Lecanemab also showed a reduction in the synaptic injury marker neurogranin in the CSF, whereas NfL levels in the CSF did not separate from the placebo over 78 weeks of treatment. Both lecanemab and donanemab showed a significant reduction in the astrocytic marker plasma glial fibrillary acidic protein (GFAP) over 78 weeks [4,92,94].

The dose of oral ALZ-801/valiltramiprosate being used in current clinical trials provides CNS concentrations that fully block the formation of neurotoxic soluble oligomers in mechanism of action studies [37]. This dose has shown the most pronounced p-tau_181_ reduction compared with the effects reported with other anti-amyloid agents, including anti-amyloid antibodies [26,58]. This is consistent with the promising clinical efficacy that has been observed in APOE4 carriers treated with ALZ-801′s active agent tramiprosate.

## 7. Targeting of Amyloid Oligomers Necessary for Efficacy in Alzheimer’s Disease

To date, the only successful Alzheimer’s disease-modifying trials have been with agents that target beta-amyloid toxicity at the early symptomatic stage, called early AD, and the selectivity for amyloid oligomers closely correlates with the clinical efficacy of anti-amyloid agents. Anti-amyloid antibodies, some of which showed positive results in Phase 3 trials, show distinct profiles of binding to soluble oligomers and protofibrils versus insoluble amyloid fibrils and plaques, profiles that correspond to their respective efficacy and safety.

Among these antibodies, lecanemab, while exhibiting the lower brain penetration, is the most selective for soluble oligomers and larger soluble amyloid aggregates called protofibrils. Aducanumab and donanemab have similar intermediate selectivity for oligomers, binding to plaque much more than to oligomers, whereas gantenerumab, which failed in Phase 3 trials, shows the weakest oligomer selectivity [9,60,63].

ALZ-801/valiltramiprosate, an oral agent that blocks the misfolding of amyloid monomers and the formation of all soluble amyloid aggregates [59], has shown positive biomarker effects and promising efficacy in APOE4 carrier early AD patients [2,26,58] without increasing the risk of ARIA [26,58]. Tramiprosate, the active anti-oligomer agent in ALZ-801, was evaluated in a Phase 3 AD study in mild and moderate AD subjects and showed significant cognitive benefits in APOE4/4 homozygotes [29,74]. In the mild AD APOE4/4 group, the effect on the Alzheimer’s Disease Assessment Scale—cognitive subscale (ADAS-cog) was 5.4 points, and effects on the functional outcomes of the Clinical Dementia Rating—Sum of Boxes (CDR-SB) and the Disability Assessment for Dementia (DAD) were also nominally significant. These findings were the basis for the pivotal APOLLOE4 Phase 3 trial to evaluate ALZ-801 in APOE4/4 homozygotes with early AD, which has completed enrollment of 325 subjects across North America and Europe and will report results in 2024.

Across anti-amyloid treatment programs, the profile of clinical efficacy parallels the order of oligomer selectivity, whereas ARIA-E risk follows the degree of plaque binding, as summarized in Table 3. Lecanemab, which is more selective to oligomers than other late-stage antibodies, provides the best efficacy with the lowest ARIA rates.

To date, the US FDA has approved two anti-amyloid antibodies for early AD patients: aducanumab and lecanemab. Aducanumab (Aduhelm) received accelerated approval in 2021 based on amyloid plaque clearance in two Phase 3 studies [3,97]. Lecanemab (Leqembi) received accelerated approval for amyloid plaque clearance, followed by traditional approval in 2023 based on positive results in a large Phase 3 trial [4,98]. A third amyloid antibody, donanemab, reported positive clinical outcomes in a Phase 2 and a Phase 3 trial [5,28]. A summary of the clinical efficacy results from the Phase 3 trials is provided in Table 4.

Among these antibodies, lecanemab has shown the most consistent efficacy across clinical outcomes in Phase 2 and 3 trials [4,27]. Aducanumab, according to data from the EMERGE Phase 3 trial, and donanemab have similar efficacy profiles [3,5,28], following their similar selectivity for amyloid species.

From a safety perspective, lecanemab has shown the lowest rate of ARIA-E and ARIA-H in the overall Phase 3 study population, while aducanumab and donanemab show higher rates, as summarized in Table 5. All three antibodies consistently show several-fold higher ARIA-E rates in APOE4/4 homozygotes than in non-carriers.

The increased risk of ARIA-E in APOE4 carriers, especially in APOE4/4 homozygotes, is likely related to their higher burden of CAA [70]. Although ARIA-E can be asymptomatic or mildly symptomatic, some patients develop serious or life-threatening events, such as confusion, encephalopathy, seizures, and status epilepticus. A fatal hemorrhagic stroke and two fatal cases of necrotizing vasculitis were reported in patients treated with lecanemab [99,100]. Three fatal ARIA cases were also reported in the donanemab Phase 3 trial [5].

The risk of ARIA-E requires frequent MRI monitoring in clinical trials, which is costly and inconvenient and may limit the utility of these drugs in clinical practice. The delivery of these antibodies as monthly or twice-monthly intravenous infusions, which may be associated with infusion hypersensitivity reactions, is burdensome to patients and caregivers and limits access to treatment.

These clinical and mechanistic data highlight the importance and promise of soluble Aβ oligomers as therapeutic targets in AD and the potential benefits of oral agent ALZ-801/valiltramiprosate, which inhibits the formation of toxic amyloid oligomers without disrupting plaques and, therefore, has demonstrated no increase in ARIA side effects associated with immunotherapies [1,2,101].

## 8. Conclusions and Future Directions

The past two decades of research have brought enormous advances to our understanding of AD pathogenesis, as well as therapeutic approaches that can lead to disease modification and clinically meaningful benefits to AD patients.

Four advances enabled these essential insights: (1) the identification of neurotoxic soluble amyloid oligomers as the upstream triggers of brain neurodegeneration and as drivers of Alzheimer’s pathogenesis, (2) the biological definition of AD based on biomarkers enabling improved diagnostic accuracy using the A/T/N criteria, (3) the application of biomarkers, in particular p-tau, GFAP, synaptic markers and brain volumetrics (MRI), for the evaluation of disease course and therapeutic efficacy in AD trials, and (4) an improved understanding of the role of APOE4 genotype in Alzheimer’s pathogenesis and its effects on efficacy and safety of anti-amyloid treatments.

To date, only agents that either block formation or preferentially clear soluble amyloid aggregates have shown clinical efficacy in AD clinical trials, suggesting that Aβ oligomers are the primary causative agents in AD pathogenesis. Novel approaches that focus on the interaction of Aβ with APOE4 may provide additional attractive targets for drug discovery.

Other neurodegenerative disorders follow the same pattern of protein dysregulation, impaired clearance, and increased aggregation, leading to neurotoxicity and loss of function. In these diseases, a normal essential brain protein starts accumulating in the brain, misfolding, and aggregating into soluble oligomers. These oligomers behave like prions, propagating and seeding further aggregation across synaptic networks.

Beta-amyloid, alpha-synuclein, tau, and TDP-43 prions form pathognomonic protein aggregates that are toxic to specific neuronal populations and cause the degeneration of neuronal networks. The destruction of brain structures by oligomer assemblies results in characteristic clinical syndromes described in Alzheimer’s disease and Parkinson’s disease, amyotrophic lateral sclerosis, progressive supranuclear palsy, and many other neurological diseases [11].

These well-conserved pathways of brain neurodegeneration offer multiple opportunities for potential disease-modifying interventions to slow the progression or even prevent the onset of clinical symptoms if administered early in the course of the pathogenic process. Therapeutic strategies to treat and prevent neurodegenerative disorders include interventions to improve the clearance of toxins from the brain and the use of agents that inhibit the misfolding of proteins, aggregation into oligomers, the toxicity of pathogenic aggregates, and prion propagation.

## Figures and Tables

**Table 1:** Biomarker-based A/T/N diagnostic framework of AD.

Biomarker	Amyloid (A)	Tau (T)	Neuronal Injury (N)
**Imaging Biomarker**	Amyloid PET	Tau PET	Volumetric MRI (hippocampal volume or cortical thickness)
**CSF or Plasma Biomarker**	Aβ42/Aβ40 ratio	P-tau_181_ P-tau_217_	Total tau or NfL (axonal injury) Neurogranin (synaptic injury)

PET imaging with amyloid or tau tracers allows the detection of pathological brain aggregates associated with AD. The ratio of Aβ42/Aβ40 is measured in either the CSF or the plasma, and a low ratio indicates AD pathology [46]. P-tau is tau phosphorylated at threonine 181 or 217 sites and is measured in the CSF or plasma [88,89,90,91]. Neurofilament light chain protein (NfL) is a marker of axonal injury measured in the CSF and plasma. Neurogranin in the CSF is a marker of synaptic injury. Aβ, beta-amyloid; AD, Alzheimer’s disease; CSF, cerebral spinal fluid; MRI, magnetic resonance imaging; PET, positron emission tomography. Note: Plasma glial fibrillary acidic protein (GFAP) is a measure of astrocyte activation and is reported to correlate with both amyloid and tau burden on PET scans [92,93].

**Table 2:** Reduction in plasma p-tau_181_ correlates with clinical efficacy of anti-amyloid antibodies.

Drug	Clinical Trials	Plasma P-tau_181_ % Reduction Annualized	CDR-SB % Clinical Benefit
**Gantenerumab** 1200 mg subcutaneously every 4 weeks	Phase 3	2–4%	6–8%
**Aducanumab** 10 mg/kg IV infusion monthly	Phase 3	9–11%	22%
**Lecanemab** 10 mg/kg IV infusion twice monthly	Phase 2, Phase 3	18–20%	26–27%
**ALZ-801/valiltramiprosate** 265 mg oral tablet twice daily	Phase 2	41%	Phase 3 ongoing

Annualized rates of reduction in plasma p-tau_181_ levels and corresponding effects on CDR-SB are shown for gantenerumab Phase 3 trials, including the open-label extension [95,96], aducanumab Phase 3 trials [3], lecanemab Phase 2 and 3 trials [4,27], and ALZ-801/valiltramiprosate Phase 2 trial [26,58]. CDR-SB, Clinical Dementia Rating—Sum of Boxes; IV, intravenously; p-tau_181_, tau phosphorylated at threonine 181.

**Table 3:** Amyloid oligomer selectivity determines clinical efficacy, while plaque clearance correlates with brain edema and microhemorrhage of anti-amyloid treatments.

Oligomer Selectivity and Pharmaceutical Profile	*Biogen* Aducanumab IV Infusion 10 mg/kg Monthly	*Eli Lilly* Donanemab IV Infusion 1400 mg Monthly	*Eisai* Lecanemab IV Infusion 10 mg/kg Twice Monthly	*Alzheon* ALZ-801/Tramiprosate Oral Tablet 265 mg Twice Daily
**Relative Selectivity for Soluble Amyloid Oligomers**	+	+	++	+++ Fully blocks formation of neurotoxic oligomers
**Brain Penetration** **Ability to Cross Blood–Brain Barrier**	Low 1.5%	Low 0.1%	Low 0.3%	High 40%
**Study Population**	Early AD All genotypes	Early AD All genotypes	Early AD All genotypes	Mild AD APOE4/4 subgroup
**Cognitive Benefit** ADAS-cog (% vs. placebo)	27%	19%	26%	125%
**Functional Benefit** CDR-SB (% vs. placebo)	22%	29%	27%	81%
**ARIA-E Brain Edema** (% in active arm)	35% 64% in APOE4/4	24% 41% in APOE4/4	13% 33% in APOE4/4	0%

Selectivity for amyloid oligomers and brain penetration determine the clinical efficacy of anti-amyloid agents. Brain edema and microhemorrhages are a result of the clearance of insoluble amyloid aggregates deposited in the vessel wall by anti-amyloid antibodies [1,2,3,4,5,28,95]. AD, Alzheimer’s disease; ADAS-cog, Alzheimer’s Disease Assessment Scale—cognitive subscale; APOE4/4, homozygosity for ε4 allele of apolipoprotein E gene; CDR-SB, Clinical Dementia Rating—Sum of Boxes; IV, intravenously. ALZ-801/tramiprosate data are from the Phase 3 tramiprosate APOE4/4 subgroup; *N* = 426 is total number of Phase 3 subjects with serial MRIs [29]. Note: donanemab clinical effects are from the overall Phase 3 study population of 1736 subjects. The (+) sign signifies the relative selectivity for soluble amyloid oligomers, with (+) and (+++) denoting lowest to highest selectivity. This is a semiquantitative way of showing that some of the agents are more selective than others: (+) for Aducanumab and Donanemab, (++) for Lecanemab and (+++) for ALZ-801.

**Table 4:** Clinical efficacy outcomes in Phase 3 trials of anti-amyloid antibodies.

Outcome Measure	Aducanumab ENGAGE * Phase 3 N~550/arm	Aducanumab EMERGE Phase 3 N~550/arm	Donanemab Phase 3 † N~850/arm	Lecanemab Phase 3 ‡ N~900/arm
**CDR-SB**	−2% (27%) *p* = NS	22% *p* = 0.012	29% *p* < 0.0001	27% *p* < 0.0001
**ADAS-cog**	11% *p* = NS	27% *p* = 0.010	19% *p* < 0.001	26% *p* < 0.001
**ADCS—iADL**	18% *p* = NS	40% *p* < 0.001	28% *p* < 0.001	37% *p* < 0.0001

Phase 3 studies were of ~18 months duration in early AD patients. The percentages shown are benefits in the active arm versus the placebo arm (A − P/P × 100). ADAS-cog, Alzheimer’s Disease Assessment Scale—cognitive subscale; ADCS-iADL, Alzheimer’s Disease Cooperative Study—Instrumental Activities of Daily Living Inventory; CDR-SB, Clinical Dementia Rating—Sum of Boxes; NS, not significant. * Aducanumab results are shown for the high-dose arm versus placebo. The ENGAGE trial effect on CDR-SB is shown for the subgroup that received dosing after protocol amendment [3]. † Donanemab Phase 3 trial TRAILBLAZER-ALZ 2 results are shown in the overall study population of 1736 subjects [5]. ‡ Lecanemab Phase 3 CLARITY AD trial [4].

**Table 5:** ARIA-E and ARIA-H rates in Phase 3 trials of anti-amyloid antibodies.

ARIA	Aducanumab ENGAGE Phase 3 N~550/arm	Aducanumab EMERGE Phase 3 N~550/arm	Donanemab Phase 3 N~850/arm	Lecanemab Phase 3 N~900/arm
**ARIA-E** **Incidence**	36% 64% in homozygotes *	35% 64% in homozygotes *	24% 41% in homozygotes	15% 33% in homozygotes
**ARIA-E** **Symptomatic**	6%	6%	6%	3%
**ARIA-H** **Incidence**	19%	20%	31%	17%

ARIA rates of anti-amyloid antibodies closely follow their affinity for insoluble aggregated amyloid and ability to clear plaques and fibrils detected on PET imaging. Aducanumab Phase 3 trials [3], donanemab Phase 3 trial [5], and lecanemab Phase 3 trial [4]. APOE4/4, homozygosity for ε4 allele of apolipoprotein E gene; ARIA, amyloid-related imagining abnormalities with edema (ARIA-E) or hemosiderin deposits (ARIA-H), which includes microhemorrhages or cortical superficial siderosis; PET, positron emission tomography. * Aducanumab ARIA-E incidence rate in APOE4/4 homozygotes is from pooled high-dose arms of both studies.

## Data Availability

Not applicable.
